# Ergonomic challenges in healthcare: mapping physical load during patient transfers using electromyographic field measurements

**DOI:** 10.3389/fpubh.2024.1459595

**Published:** 2024-11-25

**Authors:** Jonas Vinstrup, Markus Due Jakobsen, Anders Bruun Nielsen, Lars Louis Andersen

**Affiliations:** ^1^National Research Centre for the Working Environment, Copenhagen, Denmark; ^2^Department of Health Science and Technology, Aalborg University, Aalborg, Denmark

**Keywords:** healthcare, ergonomics (environmental health), electromyography, physical load, nurses

## Abstract

**Purpose:**

Work-related musculoskeletal disorders are prevalent among healthcare workers. These workers experience high rates of low-back pain; partly due to the high physical demands of patient transfers. Understanding the specific transfer scenarios that contribute to high physical loads is therefore crucial for developing strategies to improve working conditions.

**Methods:**

This study utilized electromyography to measure muscle activity in the erector spinae muscles during patient transfers, performing measurements in real-life hospital settings to identify the physical load associated with different transfer scenarios. Using linear mixed models, the 95th percentile ranks of the normalized root mean square (nRMS) values were analyzed for a range of different patient transfers.

**Results:**

The results revealed significant differences in physical load across various patient transfer scenarios. High-load activities included sitting to lying down or lying down to sitting (nRMS 32.7, 95% CI: 28.9–36.6) and lifting the upper body (32.4, 95% CI: 28.8–35.9), while low-load activities such as supporting patients while walking or standing (21.9, 95% CI: 18.6–25.1) and mobilizing in bed (19.9, 95% CI: 16.1–23.8) required less muscle activation. Moderate-load activities included bed to chair transfers (28.1, 95% CI: 24.9–31.3) and lifting the head (26.3, 95% CI: 22.7–29.9).

**Conclusion:**

Understanding the physical load associated with different patient transfer scenarios allows for better organization of work in healthcare settings. These novel findings emphasize the need for effective task allocation, rotational schedules, and the use of assistive devices to distribute physical load and reduce injury risk.

## Introduction

The prevalence of work-related musculoskeletal disorders (MSDs) among healthcare workers remains a significant concern, with current estimates suggesting alarmingly high occurrences of low-back pain (LBP) and MSDs (1, 2). Notably, nurses and nursing aides are particularly vulnerable, experiencing LBP and back injuries at rates considerably higher than other healthcare professionals as well as the general population (3–5). The work-related consequences of these prevalences are similarly profound. Previous research has found that nurses experiencing MSDs report lower levels of job satisfaction and are significantly more likely to leave their positions (6). In fact, in the cross-country survey by Aiken et al., more than half of the included healthcare workers below the age of 30 planned to leave within 1 year due to the inherent challenges of the job. Alas, the current situation within healthcare is worsened by the fact that the profession is experiencing a global shortage of nurses; one that is estimated to increase by 2030 (7). To this, it is likely that at least part of the reason is due to factors inherently related to the local working environment (e.g., high work pace, inadequacy of staff and resources, emotional exhaustion, etc.), which – aside from the aforementioned prevalence of MSDs (8) - is associated with higher ratings of perceived exertion, stress, burnout, and fatigue (9–15).

In this context, the accumulation of high physical workloads constitutes an equally recurrent and potent risk factor in the literature (16–18). Specific to the work environment of healthcare workers, a range of prospective studies have elucidated the negative consequences of high loads during patient transfers (19–21), while appropriate use of assistive devices have shown to somewhat mitigate these consequences (22–24). Following this, we recently investigated the physical load attributed to the use of different assistive devices (21, 24), and found that the ceiling-lift and intelligent bed are associated with relatively low physical load. However, while these assistive devices have both obvious and proven efficiency in decreasing physical exposure (25–27), the real-world efficacy of such interventions remains a topic of debate (28–30). Therefore, given the physically-demanding nature of the profession, it is imperative to identify individual- and contextual factors contributing to high physical loads during patient transfer. For example, while the vast majority of transfers are inherently composed of a range of small patient handlings or sub-transfers (e.g., turning the lying patient on the side, lifting the upper body and legs in order to achieve a seated position for subsequent relocation to a chair), knowledge about which types of specific transfer scenarios that are associated with high physical load is sorely lacking from the literature, and would provide valuable, practical guidelines as to how to better distribute patient transfers among the available personnel.

Therefore, to investigate the extent of which different patient transfer activities contribute to variations in the accumulated physical load among healthcare workers, we utilized objective field measurements during a wide range of different real-life patient transfers. Importantly, we did so with enough detail to be able to identify exactly when and where during the specific transfer the physical load is peaking; subsequently allowing recommendations to be made about prioritized lifting-schedules and organization of heavy work tasks.

## Methods

### Study design and participants

We have previously detailed the methods used in this study in the protocol article for this project (31), and published results from technical measurements during patient transfers with and without the use of assistive devices (21), as well as investigated the prospective associations with LBP (24). Consequently, the following sections will reference this publication while summarizing key information to provide an overview of study design and methods specific to the analyses presented herein.

The present study utilizes data from field-measurements of erector spinae muscle activity during full workdays across Danish hospitals. A total of 52 female healthcare workers (mean ± SD; age 42 ± 10y; height 167 ± 6 cm; body mass 67 ± 12 kg; work experience 15 ± 9y) from 16 different departments at five hospitals volunteered to participate in the study (Table 1). Criteria for inclusion were measurements of blood pressure < 160/100, the absence of pregnancy and progressive/life-threatening diseases as well as an estimated high number (>5) of patient transfers during the workday.

**Table 1:** Demographics of the sample population.

Variable	Mean	SD
*n*	52	
Age	42	10
Height (cm)	168	6
Body mass (kg)	67	12
Years in the profession	15	9
Blood pressure	130/83	10/8
Pain intensity (0–10)		
*Low-back*	0.64	0.95
*Neck/shoulder*	0.50	1.02
Perceived physical exertion during patient handling (0–10)	2.7	1.3

### Data collection and analyses

In short, measurements of patient transfers were performed throughout the full workday, while recording the assistive devices utilized, the number of workers participating in the transfer, as well as self-reliance (defined as the ability to perform transfers independently; rated by the healthcare worker using a 5-point Likert scale), sex and anthropometrics of the patient. The participants were instructed to perform their transfers as usual without consideration to their participation in the study. As highlighted later in the discussion section, this approach is novel in using field-measurements throughout an entire workday, accounting for all the aspects that surrounds the patient transfer scenario.

#### EMG signal sampling and analysis

Surface EMG measurements of muscle activity were recorded using wireless equipment (TeleMyo DTS Telemetry, Noraxon, AZ, United States). The sampling rate was set at 1500 Hz with a bandwidth of 10–500 Hz. The amplifier had a 16-bit A/D converter and a common mode rejection ratio > 100 dB.

**Figure 1:**
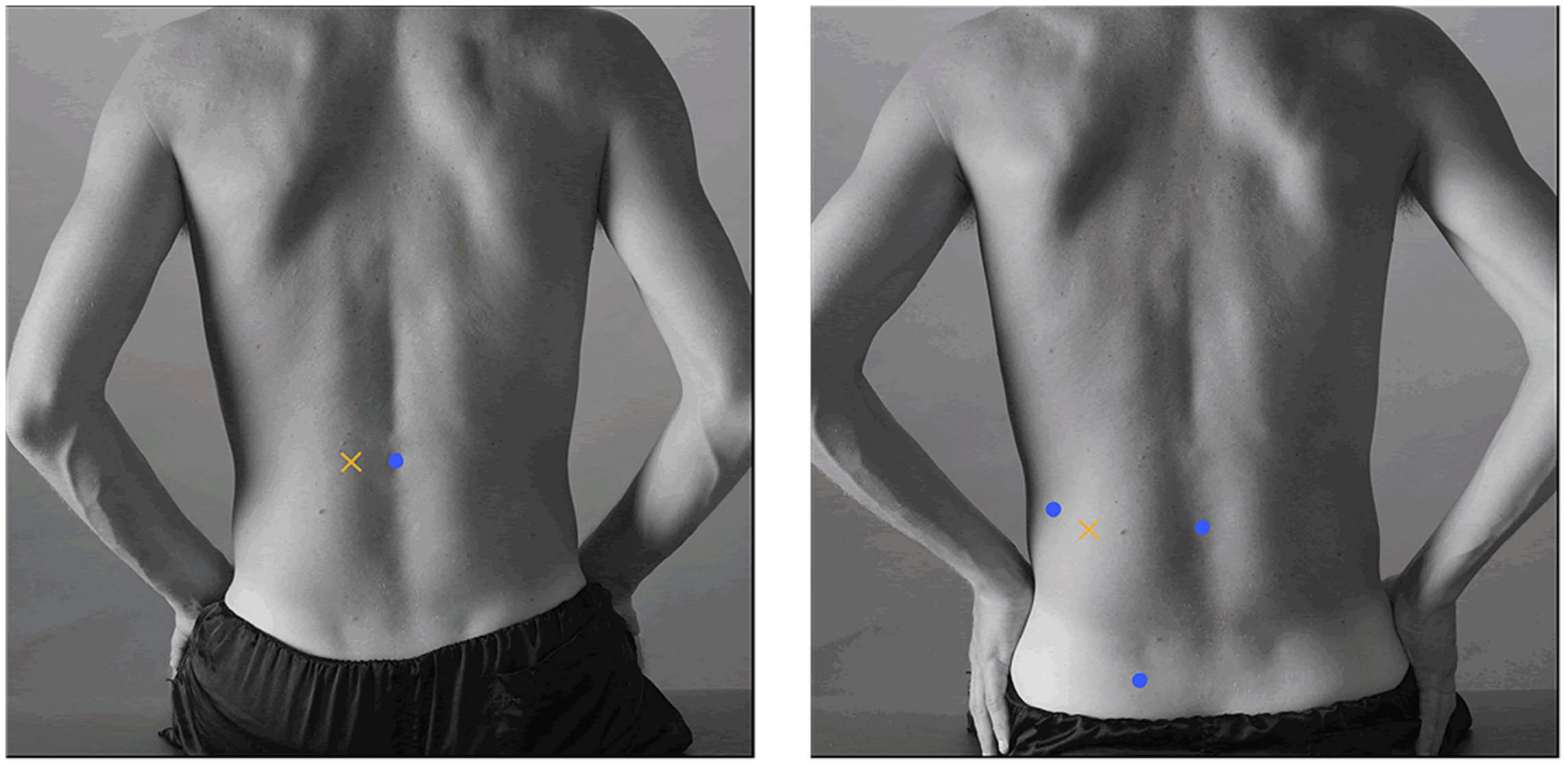
Illustration of electrode placement for the erector spinae muscles (left; longissimus, right; iliocostalis). With permission from the SENIAM group.

Prior to placing the electrodes (Blue Sensor N-00-S, Ambu A/S, Ballerup, Denmark; measuring area; 95 mm^2^, typical AC impedance; 600 ohm, combined offset instability and internal noise; <15 μV), the skin was prepared with scrubbing gel (Acqua gel, Meditec, Parma, Italy). Following the SENIAM recommendations (32), the electrodes were placed bilaterally on the erector spinae muscles with an inter-electrode distance of 20 mm (Figure 1):

1. Longissimus; two finger widths lateral from L1;
2. Iliocostalis; one finger-width medial from the line of the posterior spinae iliaca superior to the lowest point of the rib at the level of L2.

Following application of the equipment, the EMG normalization procedure consisted of maximal voluntary contractions (MVC) for the erector spinae muscles performed in the Biering-Sorensen position (33–35). The MVCs were performed twice in the morning and twice in the afternoon, with the highest recorded value used for normalization. Subsequently, all raw surface EMG signals were digitally filtered using a Butterworth fourth-order high-pass filter (10 Hz cut-off frequency), and smoothed using a root mean square (RMS) filter with a moving window (500 ms.). For each individual muscle and each patient transfer, the 95^th^ percentile rank of the smoothed RMS signal was normalized (nRMS) to the maximal moving RMS (500-ms time constant) EMG obtained during MVC. Lastly, nRMS values of the four erector spinae muscles were merged in order to obtain a larger, coherent measurement sample representing the low-back.

### Ethics

In line with the Helsinki Declaration, all participants were informed about the content of the study protocol before providing written informed consent. The information was given both written and verbally before commencement of data collection. The study was approved by the Danish National Committee on Biomedical Research Ethics (The local ethical committee of Frederiksberg and Copenhagen; H-3-2010-062) and the Danish Data Protection Agency (j.nr. 2015-41-4232).

### Statistics

Data were analyzed using linear mixed models (Proc Mixed, SAS version 9.4) with repeated measures. The 95^th^ percentile rank of the nRMS was the dependent variable and type of patient transfer was the independent variable. Analyses were controlled for age of the nurse, number of nurses, height of the nurse, body mass of the nurse, body mass and self-reliance of the patient, and the use of assistive devices. Estimates are least square means and 95% confidence intervals (95% CI) for each transfer activity.

## Results

**Figure 2:**
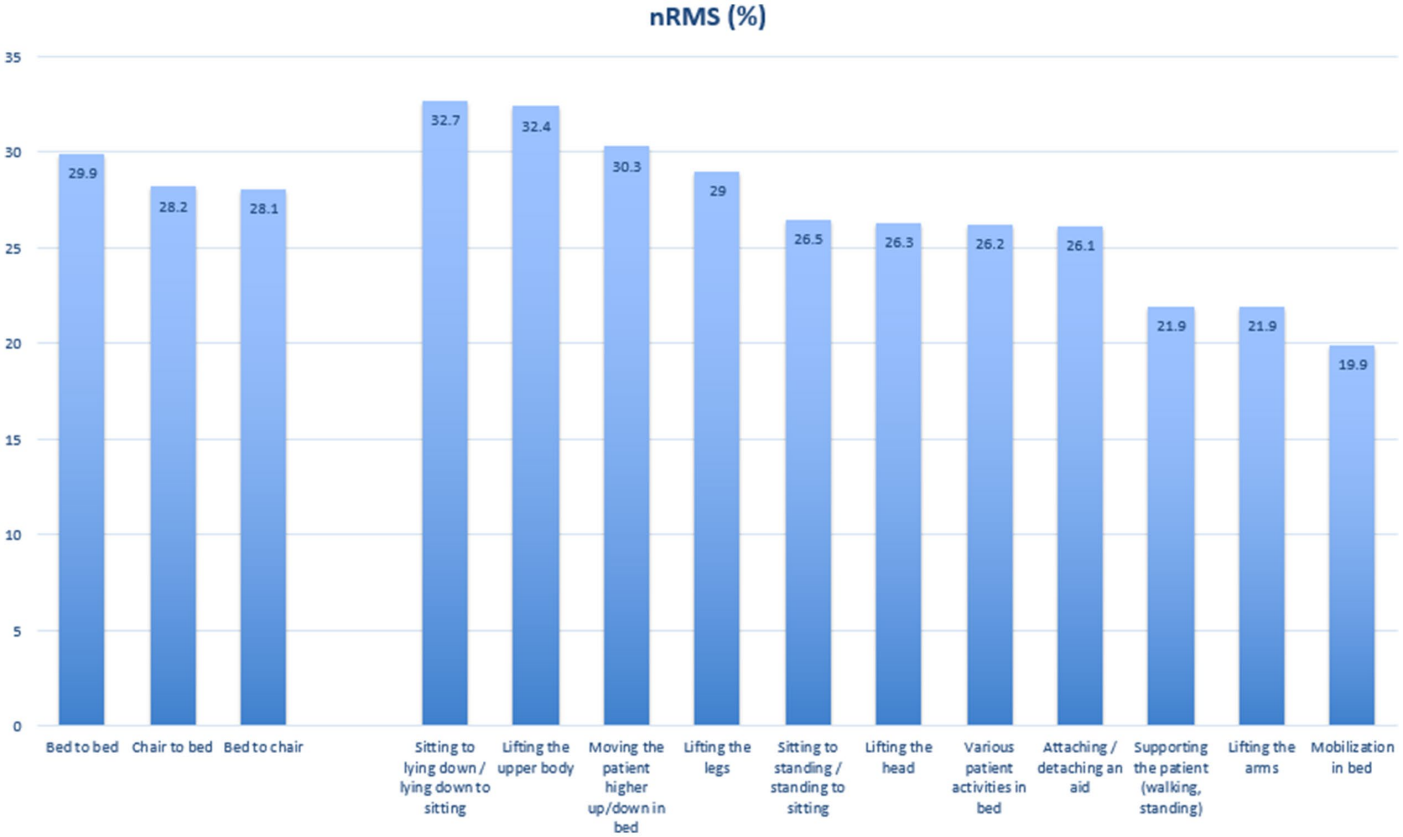
Graphical illustration of the obtained nRMS values for the erector spinae muscles during patient handlings.

Table 2 illustrates the differences in physical load during various patient transfer scenarios. For the sake of simplicity and in order to create easy-to-understand practical guidelines, these are grouped into three categories of activities based on the associated physical demand; low, moderate, and high load. Similarly, the results are graphically presented in Figure 2.

**Table 2:** Normalized EMG (nRMS) values for erectors spinae muscles during different types of patient handlings.

Activity	Mean nRMS	95% CI
Bed to bed	29.9	26.6–33.2
Chair to bed	28.2	25.0–31.4
Bed to chair	28.1	24.9–31.3
Sitting to lying down/lying down to sitting	32.7	28.9–36.6
Lifting the upper body	32.4	28.8–35.9
Moving the patient higher up/down in bed	30.3	27.0–33.6
Lifting the legs	29.0	25.8–32.2
Sitting to standing/standing to sitting	26.5	23.2–29.9
Lifting the head	26.3	22.7–29.9
Various patient activities in bed	26.2	22.9–29.6
Attaching/detaching an aid	26.1	22.9–29.3
Supporting the patient (walking, standing)	21.9	18.6–25.1
Lifting the arms	21.9	18.5–25.3
Mobilization in bed	19.9	16.1–23.8

Values are controlled for age of the nurse, number of nurses, height of the nurse, body mass of the nurse, body mass and self-reliance of the patient, and the use of assistive devices.

### Low load activities

The activities classified as low load, with mean physical load values below 25% of maximum strength capacity, require the lowest degree of low-back muscle activity. Supporting the patient while walking or standing (21.9, 95% CI: 18.6–25.1), lifting the arms (21.9, 95% CI: 18.5–25.3), and mobilization in bed (19.9, 95% CI: 16.1–23.8) fall into this category. These tasks generally involve minimal lifting of the patient, thus exerting the least strain on the healthcare worker.

### Moderate load activities

Tasks requiring moderate levels of muscle activity, with mean physical load values between 25 and 30%, include sitting to standing or standing to sitting (26.5, 95% CI: 23.2–29.9), lifting the head (26.3, 95% CI: 22.7–29.9), attaching or detaching an aid (26.1, 95% CI: 22.9–29.3), and various patient activities in bed (26.2, 95% CI: 22.9–29.6). Additionally, bed to chair (28.1, 95% CI: 24.9–31.3) and chair to bed (28.2, 95% CI: 25.0–31.4) transfers, as well as lifting the legs (29.0, 95% CI: 25.8–32.2), are considered moderate load activities.

### High load activities

High load activities, with mean physical load values exceeding 30%, demand significant physical effort. These include moving the patient higher up or down in bed (30.3, 95% CI: 27.0–33.6), sitting to lying down or lying down to sitting (32.7, 95% CI: 28.9–36.6), and lifting the upper body (32.4, 95% CI: 28.8–35.9). These tasks are the most physically demanding of the included patient transfer scenarios.

## Discussion

In summary, when investigating low-back muscle activity during range of different patient transfers, the identified low load activities include supporting patients while walking or standing, lifting the arms, and mobilization-practices in bed. Likewise, activities such as sitting to standing and lifting the head, require a moderate level of effort, and are, due to their inclusion in the majority of patient transfers, therefore likely to result in an accumulation of bodily fatigue. Similarly, high load activities, which involve moving the patient higher up or down in bed, lying down to sitting, and lifting the upper body, exhibits significant physical demands on the lower back of the healthcare worker, and hence pose the highest risk for injury. The novel findings of this study therefore emphasize the variability in physical load across different patient transfer scenarios, underscoring the necessity for strategic approaches to manage the inherent physical demands among on healthcare workers.

This inherent variability between patient transfer scenarios has obvious implications for the prevention of work-related MSDs, which, as mentioned introductorily, are highly prevalent among healthcare workers (1, 2). It is widely known that accumulation of demanding physical tasks throughout the workday increases the risk of bodily fatigue, injury, and sickness-absence (16, 17, 36, 37), and that tools or organizational practices aiming at decreasing the physical workload, alleviate these risks. For example, in the specific context of healthcare, this effect of diminishing the physical load is palpable through consistent use of assistive devices during patient transfers (19, 23, 24). However, it is also evident that healthcare workers experience a range of individual, contextual, and organizational barriers in actively using assistive devices when appropriate (38); often rendering any attempts to heighten the quality and safety of the physical work environment futile. This practical reality is, of course, not without consequence. Aside from, and partially because of, the aforementioned high prevalence of MSDs among nurses, burnout constitutes an increasingly growing problem within healthcare (39–41). Specifically, high physical workloads are known to (also) increase the incidence of burnout among this population (41, 42), as well as to hinder adequate recovery from the work shift (43). Contrastingly, a recent cohort study showed that changing to an occupation with lower physical work demands is associated with reduced risks of disability pension (44), further highlighting the need for better management and organization of physical demanding tasks during work. Considering the projected shortage of nurses and the combined implications of the studies highlighted above, the current situation and its forecast remain relatively straightforward: the healthcare occupation is in dire straits, and ameliorating the physical workload of the job seems critically necessary.

This is where the results from the present study make their much-needed entrance. Previously, it has almost solely been the results of laboratory studies illuminating the estimated load of various types of transfer, but even during these circumstances, details are sorely lacking (45–49). While the methodologies cannot be directly compared, it should be highlighted that the 3D biomechanical evaluation study by Skotte et al. report similar findings: the authors state that patient-handling tasks could classified into three groups characterized by lifting, repositioning or turning, with a corresponding gradual decrease in low-back loading (46). This is in line with the results of the present study, with high-load values for transfers involving lifting of the patient and low-load values for scenarios mainly involving repositioning in bed.

Further, the novelty of the present study lies in the fact that, aside from utilizing field-measurements during real-life transfer scenarios, the level of detail makes it possible to pinpoint exactly when the physical load is at its highest. Namely, the categorization of transfer activities based on physical demands allows for identification of low- and high load scenarios before engaging in said transfer, which enables the employment of organized lifting strategies and conferral with in-house work environment professionals. For example, when moving a patient higher up or down in bed, healthcare personnel would likely benefit from consistent use of mechanical- or ceiling mounted lifting systems, as well as adjusting the bed height to appropriate levels. For transitions between lying down and sitting/standing, workers could, when appropriate and in line with patient characteristics, employ standing aids and beds with adjustable head- and foot sections, as well as encourage the patient to use bed rails or a trapeze bar to assist in repositioning themselves. Importantly and of essential practical relevance, this means that hospitals, eldercare homes, and similar institutions performing patient transfers, are able to actively organize their daily work tasks based on a combination of the scheduled activities, patient characteristics, and physical capacity of the healthcare worker.

### Strengths and limitations

As highlighted previously, one of the primary strengths of this study is the use of real-life field measurements to assess the physical load experienced by healthcare workers during various patient transfer scenarios. Conducting the study in a naturalistic setting allows for a more accurate representation of the physical demands encountered in everyday contexts. This approach enhances the ecological validity of our findings, providing practical insights that are directly applicable to the working conditions of healthcare personnel. By capturing data in real-time, we were able to document the actual physical loads experienced by healthcare workers, and group these into load-based categories. The latter point is crucial for developing effective task-allocation strategies and ergonomic interventions to reduce the incidence of MSDs, making this study the first of its kind toward a more comprehensive understanding of the physical work environment among healthcare workers.

Limitations include the use of electromyography to measure muscle activity as a proxy for physical load, as well as focusing only on the low-back musculature. While our focus on the erector spinae muscles provides important information about low-back loads during different patient transfers and reflects the most problematic area in this population in terms of musculoskeletal disorders, other muscle groups are, of course, also involved in these tasks. Future studies would benefit from the approach of measuring EMG from multiple muscle groups to provide a fuller picture of the physical demands across different body regions. Also, while EMG is a valuable tool for assessing muscle activation and estimating physical load, the method has inherent limitations (50–52). EMG signals are known to be influenced by various factors such as electrode placement, skin impedance, and individual anatomical differences, which can introduce variability into the measurements. Additionally, EMG measures muscle activity rather than direct physical load or force, which may not fully capture the physiological strain experienced during patient transfers. Lastly, while the included sample accurately reflects the population of healthcare workers, i.e., predominately female workers, it is uncertain if the results are generalizable to male workers. However, as this study sample experience a relatively low level of pain intensity in the low-back, it is important to emphasize that the results may vary when studying a population of female healthcare workers experiencing higher levels of pain, as this is known to influence both movement patterns and preferred lifting techniques.

## Conclusion

This study emphasizes the need for organizational strategies to manage physical load in healthcare settings. By leveraging insights gained from real-life field measurements, healthcare facilities can implement targeted interventions to create a safer, more sustainable working environment for their staff. Understanding the physical load associated with different patient transfer scenarios is crucial for optimizing work- and task organization. Our findings highlight the substantial variability in physical demands across various transfer activities, underscoring the necessity for strategic management of these tasks. Effective task allocation, the implementation of rotational schedules, and the increased use of assistive devices are well-known strategies to distribute physical load more evenly among healthcare workers, and would likely not only help in reducing the risk of musculoskeletal disorders and low-back pain, but also contribute to overall job satisfaction and worker retention. Future research should focus on the long-term impacts of such interventions and their effectiveness in real-world settings, ultimately promoting safer physical working conditions, improving healthcare worker retention, and maintaining a high quality of patient care.

## Data Availability

The raw data supporting the conclusions of this article will be made available by the authors, without undue reservation.
